# *Cornus officinalis*: a potential herb for treatment of osteoporosis

**DOI:** 10.3389/fmed.2023.1289144

**Published:** 2023-12-04

**Authors:** Xinyun Tang, Yuxin Huang, Xuliang Fang, Xuanying Tong, Qian Yu, Wenbiao Zheng, Fangda Fu

**Affiliations:** ^1^Institute of Orthopaedics and Traumatology, The First Affiliated Hospital of Zhejiang Chinese Medical University (Zhejiang Provincial Hospital of Traditional Chinese Medicine), Hangzhou, China; ^2^The First Clinical Medical College, Zhejiang Chinese Medical University, Zhejiang, China; ^3^The Third Clinical Medical College, Zhejiang Chinese Medical University, Zhejiang, China; ^4^Department of Orthopedics, Taizhou Municipal Hospital, Taizhou, China

**Keywords:** osteoporosis, Cornus officinalis, kidney-tonifying herbs, bone homeostasis, effective ingredients, pharmacological mechanisms

## Abstract

Osteoporosis (OP) is a systemic metabolic skeletal disorder characterized by a decline in bone mass, bone mineral density, and deterioration of bone microstructure. It is prevalent among the elderly, particularly postmenopausal women, and poses a substantial burden to patients and society due to the high incidence of fragility fractures. Kidney-tonifying Traditional Chinese medicine (TCM) has long been utilized for OP prevention and treatment. In contrast to conventional approaches such as hormone replacement therapy, TCM offers distinct advantages such as minimal side effects, low toxicity, excellent tolerability, and suitability for long-term administration. Extensive experimental evidence supports the efficacy of kidney-tonifying TCM, exemplified by formulations based on the renowned herb *Cornus officinalis* and its bioactive constituents, including morroniside, sweroside, flavonol kaempferol, Cornuside I, in OP treatment. In this review, we provide a comprehensive elucidation of the underlying pathological principles governing OP, with particular emphasis on bone marrow mesenchymal stem cells, the homeostasis of osteogenic and osteoclastic, and the regulation of vascular and immune systems, all of which critically influence bone homeostasis. Furthermore, the therapeutic mechanisms of *Cornus officinalis*-based TCM formulations and *Cornus officinalis*-derived active constituents are discussed. In conclusion, this review aims to enhance understanding of the pharmacological mechanisms responsible for the anti-OP effects of kidney-tonifying TCM, specifically focusing on *Cornus officinalis*, and seeks to explore more efficacious and safer treatment strategies for OP.

## Introduction

Osteoporosis (OP), a global skeletal disorder often referred to as the “silent disease,” is characterized by bone mass loss and microstructure degeneration, leading to an increase in the risk of fractures and imposing a substantial socioeconomic burden (1, 2). It is estimated that the number of fractures causing by OP will reach 2.6 million in 2025, double the total number in 1990, and will reach 4.5 million by 2050 in the world (3). Moreover, increasing evidence indicates that women and older individuals are particularly vulnerable to OP and its consequences, with 1/3 of women and 1/5 of men aged 50 and above experiencing osteoporotic fractures globally (1). However, prolonged use of anti-OP medication such as denosumab, teriparatide, bisphosphonates, calcitonin, and estrogen, can result in undesirable side effects, including an increased risk of malignancy, atypical femur fractures, osteonecrosis of the jaw, and cardiovascular issues (4). The concern over these side effects and the uncertain long-term efficacy of pharmacological treatments has prompted the search for alternative medications with fewer adverse events, low toxicity, high efficacy, and good tolerability.

An increasing number of researchers are exploring Traditional Chinese Medicine (TCM) as an alternative treatment for OP due to its fewer adverse events and long-term safety profile (5). *Cornus officinalis* (known as Shanzhuyu in Chinese), an ingredient commonly found in TCM formulas for bone-related diseases such as Zuo Gui Pill (ZGP) (6, 7), You Gui Pill (YGP) (7), and Liuwei Dihuang Pill (LWDHP) (8), all of which are known for its kidney-nourishing properties, exerts beneficial effects in the prevention and treatment of OP by alleviating common symptoms experienced by individuals with OP such as lumbar and knee discomfort (9, 10). Furthermore, recent research has highlighted the therapeutic potential of certain monomeric components derived from *Cornus officinalis*, independent of its inclusion in TCM formulations including flavonoids, tannins, iridoids, organic acids, polysaccharides, and lignans (11). Among them, gallic acid, morroniside, loganin, sweroside, quercetin, notoginsenoside R1, cornuside I, kaempferol, and 5-HMF, extracted from *Cornus officinalis*, may play a crucial role in OP treatment (12).

In this review, we comprehensively summarize the research on *Cornus officinalis* in the context of OP, focusing primarily on its mechanisms of action involving bone homeostasis, immunomodulation, vascularity, and bone microarchitecture, thus providing a better understanding of the therapeutic role played by *Cornus officinalis* in the pathological process of OP and its potential clinical application.

## Pathomechanism of OP

OP is a common metabolic bone disease associated with a variety of factors such as bone homeostasis, immune mechanisms, vascular changes, estrogen deficiency, mechanical stress, and the nervous system. In this section, we will discuss the pathological of OP related to these factors.

### Bone homeostasis

Several key cells in bone tissue, such as bone mesenchymal stem cells (BMSC), osteoblast (OB), and osteoclast (OC), play critical roles in bone remodeling, including bone formation and bone resorption (13). Particularly, BMSC can mainly differentiate into adipocytes and OB in bone, to play an important role in the regulation of normal bone homeostasis (14). The capacity of BMSC from OP patients to differentiate into OB is lower than that in healthy individuals (14). The shift in preferential differentiation of MSCs from OB to adipocytes accompanied by reduced bone mineral density (BMD) can contribute to OP progression (15). OB secrete various components of osteoid, such as collagen I, alkaline phosphatase (ALP), osteopontin (OPN), and osteocalcin (OCN), which then mineralize to form mature bone (16, 17). Additionally, precursor OC are enlisted and attached to the bone matrix, subsequently undergoing further differentiation into mature OC, which can release acids and lytic enzymes that facilitate the degradation of the bone matrix and absorption of aging and damaged bone tissue (18, 19). As evidenced by disturbed bone homeostasis, altered bone microstructure, and reduced bone strength, OP arises from the imbalance of bone formation and bone resorption, resulting from excessive absorption by OC or impaired generation of OB (17, 20).

Moreover, various regulatory factors and signaling pathways impact the activity of BMSC, OB, and OC, thus governing the process of bone resorption and formation processes. Significant roles are played by signaling pathways such as Wnt/β-catenin, bone morphogenetic proteins (BMP)-Smad, Hedgehog, receptor activator of nuclear factor-B ligand (RANKL)/receptor activator of nuclear factor-B (RANK)/osteoprotegerin (OPG), along with several regulatory factors. Notably, the canonical Wnt/β-catenin signaling pathway has emerged as a crucial regulator of bone formation, promoting the osteogenic process, preventing apoptosis of OB precursors, facilitating OB differentiation and inhibiting BMSC differentiation into adipocytes (21, 22). Conversely, inhibiting Wnt pathway impedes bone formation, rendering individuals more susceptible to early-onset OP and osteogenesis imperfecta (13). Similarly, activation of Hedgehog signaling pathway promotes the differentiation of BMSC into OB rather than adipocytes by upregulating Runx-2 expression, thereby enhancing bone formation (23, 24). Moreover, specific BMP and canonical TGF-β positively regulate osteogenic activity by phosphorylating downstream Smad proteins, thereby influencing the balance between OB-mediated bone formation and OC-mediated bone resorption (23, 25, 26).

Furthermore, the RANKL/RANK/OPG signaling pathway represents the most extensively studied pathway concerning OC differentiation and activity. OB release RANKL, which binds to RANK, a specific receptor on the surface of OC, triggering the transcription of downstream factors, such as c-FOS, NFATc1, tartrate-resistant acid phosphatase (TRAP), and cathepsin K (CTSK), ultimately leading to the differentiation and activation of OC (27). Meanwhile, OPG, which is also secreted by OB, competitively binds to RANK, suppressing OC activity and safeguarding bones against excessive resorption (28, 29).

The role of Notch signaling pathway in bone remodeling relies on the type of Notch receptor involved: Notch 1 fosters increased OPG production and decreased sclerostin, exerting osteoprotective effects by inhibiting OC formation and bone resorption, while Notch2 promotes osteoclastogenesis and enhances bone resorption by stimulating RANKL expression (30). In summary, OP arises from an imbalance between bone formation and bone resorption within bone homeostasis, stemming from the dysregulation of multiple signaling pathways.

**Figure 1:**
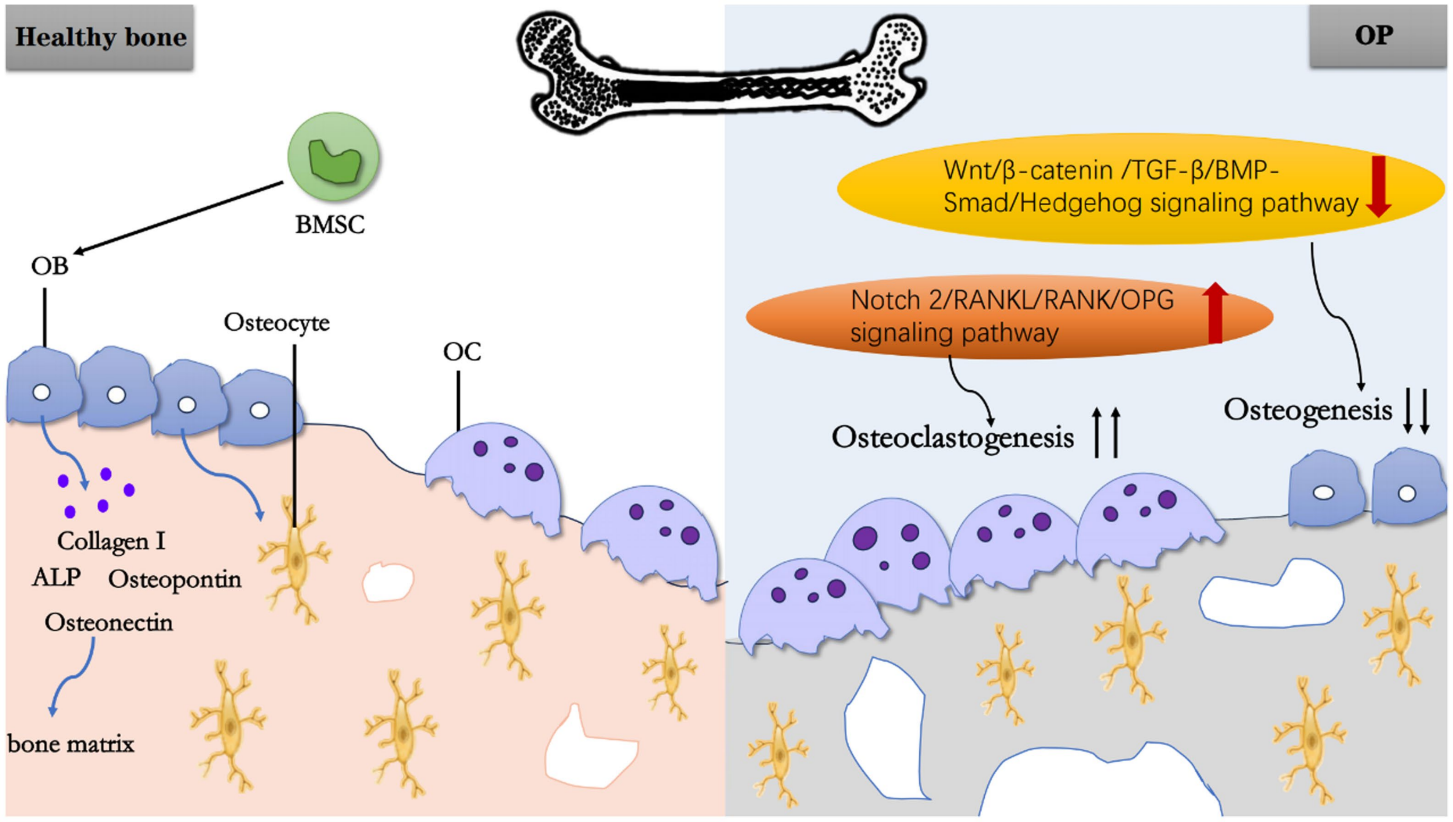
Schematic working model of OP. In a normal physiological state of healthy bone, BMSC can differentiate into OB, and OB further mature into osteocytes. Meanwhile, OB secretes Collagen I, ALP, OPN, Osteonectin, and other substances to constitute bone matrix. Under pathological state, the upregulation of Notch 2/RANKU/RANK/OPG signaling pathway promoting OC generation and the downregulation of Wnt/β-catenin/TGF-B/BMP-Smad/Hedgehog signaling pathway inhibiting osteogenesis, ultimately lead to OP.

In summary, BMSC and OB play roles in bone formation, while OC affect bone resorption. Together, they mediate OP through different factors (Figure 1).

### Vasculature

The vascular system in bones contributes to the supply of oxygen, nutrients, hormones, growth factors, and neurotransmitters necessary for the normal growth, development, regeneration, and remodeling of bone. Emerging evidence reveals that the metabolic imbalance in the bone microenvironment caused by blood vessel supply impairment, along with the imbalance of vascular calcium, phosphorus and glucose metabolism, is intricately associated with the pathological mechanisms of OP (31). For instance, adjustment of the number and size of blood vessels along the flow has confirmed that the skeletal system occupies 10 and 15% of the total cardiac output, which is crucial for skeletal system health. And insufficient blood flow can lead to delayed bone repair and other low bone mass diseases due to impaired osteogenesis (32, 33). Moreover, a female patient with a rare Hajdu-Cheney syndrome causing periarticular OP showed reduced the height and density of blood vessels in all affected fingers (34). Triggering the activation of the HIF signaling pathway in OB could prevent the reduction of blood vessels in the bone marrow of postmenopausal OP patients to prevent bone loss (35). Another study showed that supplementation of ribonuclease-rich lactoferrin promotes new vessels formation, achieving a significant reduction in bone resorption and an increase in bone formation to restore bone homeostasis (36).

Blood vessels inside bones are generally identified two types: The type H vessels with high expression of CD31 and endomucin mainly localized in the vicinity of the chondro–osseous junction, and the type L vessels with low expression of CD31 and endomucin mainly localized in the diaphysis (37). The number of Type L vessels, which does not change over time, are basically not involved in bone metabolism (37). Contrary to type L vessels, type H vessels maintain OB around blood vessels, which has been proved to be an important carrier of to induce angiogenesis and bone formation (38–40). Researches indicate a corresponding reduction in the number of type H blood vessels in aged OP mice, ovariectomized (OVX) induced OP mice, as well as in elderly and OP patients, accompanied by loss of bone precursor cells (37–40). In turn, promoting the maturation of Type H vessels by activating Notch signaling can regulate the differentiation of perivascular osteoprogenitor cells and accelerate osteogenesis (41).

The levels of calcium, phosphorus, and glucose in blood vessels, as well as their metabolic balance, are also closely related to OP. Studies have observed significant decreases in BMD, osteoclastic strength, serum ALP, and calcium and phosphorus in the OP model after ovariectomy (42–46). In addition, abnormal calcium loss of bone tissue, accompanied by calcium deposits within blood vessels causing vascular calcification, contributes to the pathogenesis of OP, known as the “calcium paradox” (47, 48). The aggravation of vascular calcium deposition obstructs nutrient supply to bone tissue and further promotes the pathogenesis of OP. On the other hand, output venous vessels in Haversian and Volkmann ducts allow immune cells to migrate from the basement membrane to the deeper layers of dense bone, becoming a site for mineral reabsorption during OP (49). Furthermore, abnormal blood glucose levels downregulate PI3K-AKT signaling pathway to inhibit OB activity and promote OC activity in the trabecular bone region with reduced serum level of OCN and ALP (50, 51). All these findings underscore the close relationship between blood vessels and the pathological process of OP, particularly the number of type H vessels and capillaries, bone blood flow and vascular calcium deposition besides vascular calcium, phosphorus and glucose metabolism (31, 34–36, 48, 49).

In conclusion, activating neovascularization, promoting bone blood flow, and balancing calcium level between blood vessels and bone tissue are therapeutic strategies for OP management.

### Immune system

Osteoimmunology is an interdisciplinary field arising from mounting evidence of the close relationship between the immune system and bone metabolism (52–54). Relevant studies have shown that many immune cells in the bone system, including T lymphocytes, B lymphocytes and macrophages, affect bone cells in the bone system directly or indirectly through the secretion of mediators by immune cells such as OPG/RANKL, COX-2, interleukins, and tumor necrosis factor (TNF) (52, 54–64).

Th2 lymphocytes, through the release of IL-4 and IL-13, act to prevent the formation of OC by downregulating prostaglandin dependent on COX-2, thereby suppressing bone resorption (54, 56). Conversely, Th17 lymphocytes, as the main source of IL-17, promote osteoclastic differentiation *in vitro* and the generation of RANKL, resulting in bone loss in mice with primary hyperparathyroidism (57–59). Meanwhile, IL-17 also promotes the early differentiation of OB by increasing the expression of ALP, RUNX2, OCN, and OPG (61–63). Besides, the upregulation of TNF-α expression in T-lymphocytes, under the regulation of RANKL, promotes the apoptosis of OB (60, 64).

The B lymphocytes, on the other hand, reduce OB differentiation by acting through CCL3 and TNF, which target ERK and NF-κB signaling pathways (65). In addition, B lymphocytes secrete various cytokines that play a dual role in OC: On one hand, they produce IL-7 (66), RANK (67), and approximately half of the total OPG (68) in the bone marrow, suppressing OC activation; On the other hand, B lymphocytes secrete G-CSF and RANKL under inflammatory conditions, promoting the differentiation and proliferation of OC, thus leading to bone resorption (69). Surprisingly, IL-18, initially considered an up-regulator of OPG that inhibits osteoclastogenesis, was subsequently found to increase the expression of RANKL on T lymphocytes, ultimately promoting bone mass loss (70).

Macrophages presented in bones are known as various populations: bone marrow macrophages (BMMs), OC, and osteal macrophages (71), all of which can categorized into two phenotypes—M1 (inflammatory phenotype) and M2 (reparative phenotype)—playing different roles in bone homeostasis. M1 macrophages, considered as precursors of OC (72), polarize after stimulation by pro-inflammatory cytokines IL-6, TNF-α, and IFN-γ (73), triggering osteoclastogenesis and subsequent bone destruction (74). Interestingly, RANKL-induced M1 macrophages contribute to the expression of *OPN* and *RUNX2* in BMSC, inducing osteogenesis as a contrary effect (75). Conversely, M2 macrophages polarize under stimulation by anti-inflammatory cytokines such as IL-4 and IL-13, and stimulate MSCs or pre-osteoblastic cells to differentiate into OB, promoting bone formation. Moreover, increased transition from M1 to M2 macrophages enhances this trend (53, 73, 76). Hence, regulating the ratio of M1/M2 macrophages holds the potential therapeutic effect of anti-OP.

### Other factors

Many other factors, including nervous system, mechanical stress, estrogen, and oxidative stress, contributing to OP based on available data. To maintain proper bone balance, the nervous system enters mature bones, regulates blood flow and metabolism, and secretes neurotransmitters (77). Neuropeptide-Y (NPY), a classic neuronal regulator of energy homeostasis, directly inhibits BMSC proliferation and OB differentiation through the Y1 receptor on the surface of BMSC or OB and the Y2 receptor in hypothalamus, thereby suppressing bone formation and leading to OP (78–81). Moreover, mechanical stress also impacts OP, preventing osteoporotic bone loss through the Pl3k/Akt signaling and erythropoiesis (82). Estrogen, in order to protect the bones, inhibits OB apoptosis and OC formation by reducing the expression of RANKL, which also promotes the apoptosis of OC (83), which contributes to an increase in the incidence of OP among postmenopausal women. Furthermore, increased oxidative stress raises TNF-α levels in serum while reducing Sirtuin 6 (Sirt6) expression in long bones, promoting NF-κB acetylation as well as CTSK over-expression and activation (84), consequently leading to bone destruction (85, 86).

## Anti-OP effects of *Cornus officinalis* and effective ingredients or Chinese formulations

### BMSC and OB are the therapeutic targets of *Cornus officinalis* and its active ingredients or compounds to exert an anti-OP role

*Cornus officinalis* has been traditionally employed in East Asia for the treatment of OP. This botanical resource boasts abundant active ingredients that exert diverse effects on OP by modulating the proliferation and differentiation capacity of BMSC, promoting osteogenic differentiation, and ameliorating the OP phenotype.

The aforementioned findings, presented in Table 1, support the notion that promoting BMSC proliferation and osteogenic differentiation, inhibiting lipogenic differentiation, enhancing osteogenesis related protein such as BMP2, Osx, RUNX2, ALP, OPN, OCN by regulating Wnt/β-catenin, BMP, PI3K/AKT/mTOR, AMPK, JNK, ERK, NF-κB signaling pathways represent promising therapeutic strategies for the treatment of OP mediated by the efficacious components of *Cornus officinalis*.

**Table 1:** Active ingredients of *Cornus officinalis* for BMSC and OB.

Sorts of compounds	Active constituents	Results	References
Flavonoids	Quercetin	Enhances osteoblastogenesis while inhibiting adipogenic differentiation through Wnt/β-catenin, BMP, AMPK, JNK, and ERK signaling pathways	(87–91)
		Restores impaired BMSC function and activity induced by TNF-α by inhibiting NF-κB activation and β-catenin degradation	(92)
	Kaempferol	Reduces apoptosis induced by LPS, stimulates MSCs proliferation, and regulates non-coding RNAs, including miR-124-3p and miR-10a-3p, to control MSCs differentiation toward osteogenic lineages, promoting bone formation	(93, 94)
		Promotes OB autophagy while decreasing OB apoptosis, elevates the expression of osteogenic markers, including ALP, OSX, COL-1, OCN, and OPN, and facilitates osteoid mineralization and calcium deposition, resulting in an increase in calcium nodules	(95)
Iridoids	Sweroside	Enhances osteogenic differentiation by activating the mTOR1/PS6 signaling pathway, resulting in upregulation of OCN, Runx2, and Osx expression in osteoporotic mice BMSC and increased mineralized nodules formulation	(96, 97)
		Contributes to considerably higher levels of BMP2, RUNX2, ALP, OPN, and bone sialoprotein-1 (BSPH1) in OVX mice, along with increased bone matrix production	(98)
	Morroniside	Boosts BMSC proliferation *in vitro*, counteracts BMSC dysfunction and impaired osteogenic differentiation and bone loss induced by high glucose via downregulating the AGE-RAGE pathway	(99, 100)
		Interacts with sodium-glucose cotransporter 2 and adenosine A2AR to improve precursor cell viability (MC3T3) and promote proliferation	(101, 102)
		Activates PI3K/AKt/mTOR pathway, leading to Beclin1-and Atg13-dependent autophagy, facilitating the transformation of MC3T3-E1 cells into mature OB	(103, 104)
	Cornuside I	Increases ALP expression and calcium deposition, while stimulating MSCs proliferation through the activation of the PI3K/AKT signaling pathway to enhance osteogenic differentiation	(105)
	Notoginsenoside R1	Enhances migration and differentiation of human adipose-derived MSCs into OB, upregulating osteogenesis marker expression, such as ALP and OCN	(106)

### OC is another therapeutic targets of *Cornus officinalis* and its active ingredients or compounds to exert an anti-OP role

OC is bone-resorbing cell that degrades bone through acid secretion and the release of proteolytic enzymes (107). *Cornus officinalis* processes the ability to restrict the differentiation of bone marrow-derived macrophages (BMMs) into OC, and suppress the translation and genetic transcription of OC-associated markers. These anti-OP effects are achieved via the active ingredients of *Cornus officinalis* (Table 2) (119).

**Table 2:** Active ingredients of *Cornus officinalis* for OC.

Sorts of compounds	Active constituents	Results	References
Flavonoids	Quercetin	Reverses increased OC in OVX rats by promoting OC apoptosis and autophagy, involving the MAPK pathway activation	(46, 108)
		Inhibits osteoclastic progenitor cell differentiation, disrupts the actin ring of mature OC, and exerts anti-OP activity while counteracting OC function	(109, 110)
		Prevents the increase of OC-promoting factors RANKL, TNF-α, IL-6, and IL-8 in RA-fibroblasts-like synoviocytes (RA-FLS) induced by proinflammatory factor IL-17	(109)
	Kaempferol	Downregulates elevated bone turnover in OVX rats, reduces the number of TRAP-positive multinucleate cells, and suppresses the transcriptional expression of OC-related markers such as c-Fos, NFATc1 in RANKL-induced RAW264.7 cells (precursor cells of OC), thereby inhibiting osteoclastogenesis	(111–113)
iridoids	Loganin	Restrains OC precursor cell differentiation in the OB–OC co-culture system, decreases TRAP activity (a molecular marker of OC number and bone resorption)	(114, 115)
	Morroniside	Suppresses TRACP enzyme activity and the expression of OC-related genes, exhibiting therapeutic potential in the treatment of OVX-induced OP in rats by inhibiting osteoclastic differentiation.	(116)
	Notoginsenoside R1	Inhibits RANKL-induced mitogen-activated protein kinases (MAPK) signaling pathway activation and subsequent OC production *in vitro*	(117)
phenolic acid	Gallic acid	Inhibits AKT, ERK, and JNK signaling pathways to reduce the expression of NFATc1 and CTSK, thus suppressing OC differentiation, reducing bone loss in the OVX-induced OP model, demonstrating prophylactic and therapeutic effects on OP	(118)

In conclusion, *Cornus officinalis* and its active constituents modulate the function, activity, and quantity of OC by inhibiting MAPK, AKT, ERK, JNK signaling pathways to reduce the expression of OC-related proteins RANKL, c-Fos, NFATc1 and CTSK, thus inhibit bone resorption. These novel findings designate *Cornus officinalis* and its active constituents as potential therapeutic anti-OC targets for the treatment of OP.

### The vascular system is one of the therapeutic targets of *Cornus officinalis* and its active ingredients or compounds exert an anti-OP role

Angiogenesis, nutritional support function, and the metabolism of blood calcium, phosphorus, and glucose are directly related to bone development and regeneration. In the context of bone diseases, vascular function is often impaired, accompanied by metabolic imbalances (42–46, 50, 51). An increasing body of evidence demonstrates the significant role of *Cornus officinalis*, its compounds, and active ingredients in addressing this issue. The following Table 3 elaborates a detailed account of their respective targets in the prevention of OP.

**Table 3:** Active ingredients of *Cornus officinalis* targeting vasculature system.

Sorts of compounds	Active constituents	Results	References
Flavonoids	Quercetin	Increases blood calcium and phosphorus contents, regulates autophagy and apoptosis of bone cells, thus preventing OP	(44, 46)
		Improves serum calcium, phosphorus, and other biochemical indexes, as well as the thickness, length, and density of the femur, and the tensile strength of the osteoporotic femur	(43)
		Increases bone turnover markers (serum ALP, OCN and urinary calcium, phosphorus, creatinine), HIF-1α gene expression, and NF-κB levels, while decreasing vascular endothelial growth factor (VEGF) and β-catenin expression	(42)
		Elevates the levels of OCN expression and ALP activity in serum, as well as urinary deoxypyridine base in diabetic rats	(50)
Iridoids	Morroniside	Upregulates the expression of CD31 and VEGFA in mice with myocardial infarction	(120)
		Upregulates the expression of Ang-1 and Tie-2 in rats with cerebral ischemia/reperfusion	(121)
Phenolic acid	Gallic acid	Regulates estrogen and improves calcium and phosphorus levels in the blood	(45)

These findings suggest that *Cornus officinalis* and its active component and compounds hold potential as a therapeutic option for OP prevention by promoting angiogenesis, enhancing bone blood flow through regulation of HIF-1α, CD31, VEGF and its receptors, and balancing calcium, phosphorus and glucose metabolism between blood vessels and bone tissues (122). Based on the aforementioned experimental data, *Cornus officinalis*, in conjunction with its formula and monomer compounds, offers potential advantages in improving blood vessels in bones and thus playing a role in the management of OP.

### The immune system is one of the therapeutic target of *Cornus officinalis* and its active ingredients or compounds exert an anti-OP role

It has been found that several kinds of immune cells can interact with OB and OC to combat OP (52). As mentioned above (the immune system part), immune cells in bone system, including T lymphocytes, B lymphocytes and macrophage, can participate in the regulation of differentiation into OB or OC by the secretion of inflammatory factors such as interleukins and TNF. Therefore, improving the inflammatory microenvironment may have the potential to regulate the function of immune cells, promote BMSC differentiation into OB and inhibit the mature of OC precursor. Previous research has indicated that active ingredients in *Cornus officinalis*, such as 5-HMF, Cornuside, loganin, and sweroside, possess anti-inflammatory properties, however, it remains uncertain whether these substances also serve as preventatives for OP (123–126). In this context, a recent study has discovered that kaempferol may suppress the upregulation of proinflammatory cytokines induced by LPS in BMSC, promote the production of anti-inflammatory factors, and inhibit the process of osteoclastogenesis and bone resorption induced by proinflammatory factor IL-1β (94). Other studies also showed that quercetin, loganin or morroniside could enhanced the M2 macrophage polarization by targeting NF-κB and Nrf2 signaling pathways indicating potential ability to promote osteoblastic differentiation and inhibit osteoclastic differentiation (127–129). Consequently, we conclude that *Cornus officinalis* and its active ingredients represent a potential therapeutic class with anti-inflammatory properties that can inhibit the progression of OP.

### Other factors are the other therapeutic targets of *Cornus officinalis* and its active ingredients or compounds to exert an anti-OP role

Oxidative stress and estrogen play important roles in maintaining bone balance, and abnormal expression of these factors leads to OP (83–86). Numerous reports have confirmed the significant effects of the active ingredients and compounds in *Cornus officinalis* for the treatment of OP. Table 4 elaborates on their respective targets in treating OP.

**Table 4:** The active ingredients of *Cornus officinalis* for other factors.

Sorts of compounds	Active constituents	Results	References
Flavonoids	Quercetin	Blocks the oxidative stress of chondrocytes, reduces apoptosis, NLRP3-mediated pyroptosis and ECM degradation	(130)
	Kaempferol	Phosphorylates ER, activates downstream ALP, Runx-2, OSX, COL1, OCN, and osteonectin, thus inhibiting OC differentiation but inducing OC apoptosis, and preventing OB apoptosis	(131)
	Notoginsenoside R1	Suppresses oxidative stress of MC3T3-E1 cells by blocking JNK pathway, thereby restoring the ability of osteogenic differentiation	(132)
		Binds to estrogen receptor as a phytoestrogen, promoting the transcription of COL1, osteonectin, OCN, Runx2, and osterix, thereby facilitating osteogenic differentiation and mineralization	(133)

These results suggest that *Cornus officinalis*, along with its active components, can inhibit OB apoptosis and OC formation by suppressing oxidative stress response and binding estrogen receptors, which aids in promoting OB differentiation. Based on the above findings, both *Cornus officinalis* and its active components offer potential advantages in maintaining bone balance and thus taking an important part in the treatment and prevention of OP.

### An anti-OP effect exerted by *Cornus officinalis*-containing formulations

*Cornus officinalis* is a constituent of many TCM formulations such as ZGP, YGP, LWDHP, Bu-Shen-Tong-Luo decoction (BSTLD), all of which are known for their kidney-nourishing properties, and extensive evidence supports the favorable impact of these formulations in the prevention and treatment of OP (Table 5) (33, 51, 134–138).

**Table 5:** The *Cornus officinalis*-containing formulations for anti-OP effect.

TCM formula	Model	Dosage and duration	Results	References
LWDHP	OVX-treated SD rats	100 g/day for 12 weeks	Stimulates osteogenetic process by activating the Wnt/β-catenin signaling pathway	(134)
	Citrate buffer-induced diabetic mice	1.8 or 3.6, or 5.4 g/kg/day for 12 weeks	Improves BMD, BV, bone microstructure, maximum load, and bending resistance in the femurs of osteoporotic rats	(135)
ZGP	OVX-treated rats	32 g/kg/day for 12 weeks	Reverses the Th17/Treg ratio, leading to increased BMD and inhibition of bone loss in OVX mice	(136)
	OVX-treated SD rats	2.3 or 4.6 g/kg/day for 12 weeks	By combining with anti-OP medicines, ZGP treatment significantly reduces bone resorption markers such as TRACP, TRACP-5b, urine oxidative deamino acid/creatinine ratio D-Pyr/Cr, and β-cross-linked C-terminal type 1 collagen	(137)
	glucocorticoid-induced SD rats	62.3 g/kg/day for 1 month	Modifies the orderly arrangement of bone trabecular compositions and bone microarchitecture, intensifies bone mechanics, and substantially increases BMD in the lumbar spine	(138)
		3.8 g/kg/day for 6 weeks	In combination with ED-71, it reduces blood glucose levels in diabetic mice and promotes osteogenic differentiation through the PI3K-AKT signaling pathway	(51)
BSTLD	OVX-treated rats	6 or 12 g/kg/day for 12 weeks	Increases blood perfusion in the bone marrow cavity	(33)

These results suggest that *Cornus officinalis*-containing formulations could mainly improve BMD and bone microstructure, stimulate osteogenetic process, increases blood perfusion in bone marrow by reversing the Th17/Treg ratio or targeting PI3K-AKT and Wnt/β-catenin signaling pathway. Based on the above findings, *Cornus officinalis*-containing formulations offer potential advantage in promoting osteogenesis in the treatment and prevention of OP.

## Conclusion and perspectives

**Figure 2:**
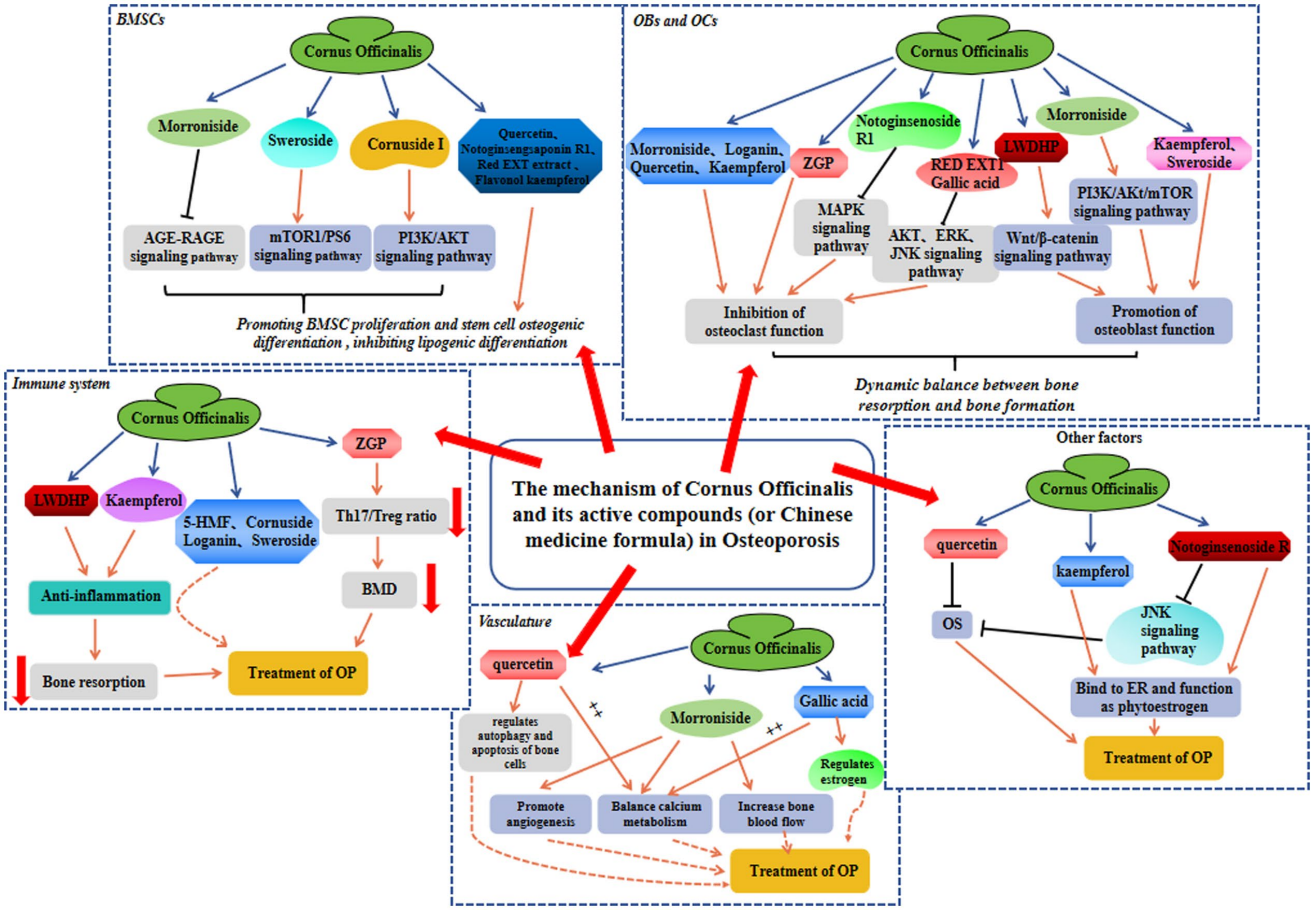
The protective mechanism involved in *Cornus officinalis* against OP in BMSC, OB and OC, immune system, vasculature, and other factors.

OP is a skeletal condition characterized by reduced BMD and compromised trabecular bone structure, which significantly increases the likelihood of fractures and imposes substantial physical and financial burdens. Within TCM application, *Cornus officinalis* is widely employed for OP treatment. Both preclinical and clinical investigations have demonstrated the effectiveness of the chemical constituents and associated formulations of *Cornus officinalis* in preventing OP, which efficacy is attributed to various mechanisms, including the modulation of bone homeostasis, promotion of angiogenesis, anti-inflammatory effects, and regulation of the immune system, etc (Figure 2). Therefore, phytochemicals from *Cornus officinalis* possess significant potential for the development of novel anti-osteoporotic medications. Herein, we provide a comprehensive review of the role of *Cornus officinalis* in multiple anti-OP mechanisms, which aligns with the multifactorial nature of OP’s etiology and surpasses the traditional model of single drug targeting single aspects of medicine. By thoroughly investigating the therapeutic properties of *Cornus officinalis* in the context of OP treatment, our aim is to enhance our understanding of TCM’s underlying mechanisms in addressing this condition. This endeavor is expected to greatly contribute to the advancement of more efficacious pharmaceutical interventions for OP. Thus, systematic data mining of the existing *Cornus officinalis* database can undoubtedly aid in the drug discovery process by identifying safe candidates.

While several compounds, such as quercetin and kaempferol, extracted from *Cornus officinalis*, have been extensively studied in relation to OP, further investigation is necessary to explore the potential effects of sweroside, notoginsenoside R1, cornuside I, morroniside, and loganin. Additionally, it is essential to identify the active components of *Cornus officinalis* through comprehensive investigations. Moreover, limitations exist in the current use of animal models for OP research. The majority of *in vivo* studies employ rodent models, which possess dissimilar cortical-to-cancellous bone ratios compared to humans, and inter-species cellular differences result in deficiencies in both *in vivo* and *in vitro* experiments, which must be further corroborated using in mammalian or primate models (139, 140). Furthermore, the existing research primarily focuses on the efficacy of *Cornus officinalis* in preclinical experiments, necessitating the need for clinical trials to substantiate its effectiveness and safety.

## Author contributions

XTa: Writing – original draft. YH: Writing – original draft. XF: Writing – original draft. XTo: Writing – original draft. QY: Writing – original draft. WZ: Writing – review & editing. FF: Writing – review & editing.
